# The Identification of Scientific Communities and Their Approach to Worldwide Malaria Research

**DOI:** 10.3390/ijerph15122703

**Published:** 2018-11-30

**Authors:** José Antonio Garrido-Cardenas, Francisco Manzano-Agugliaro, Lilia González-Cerón, Francisco Gil-Montoya, Alfredo Alcayde-Garcia, Nuria Novas, Concepción Mesa-Valle

**Affiliations:** 1 Department of Biology and Geology, University of Almeria, 04120 Almeria, Spain; jcardena@ual.es (J.A.G.-C.); cmesa@ual.es (C.M.-V.); 2 Department of Engineering, University of Almeria, CeiA3. 04120 Almeria, Spain; pagilm@ual.es (F.G.-M.); aalcayde@ual.es (A.A.-G.); nnovas@ual.es (N.N.); 3 Regional Center for Public Health Research, National Institute of Public Health, Tapachula 30700, Chiapas, Mexico; lgonzal@insp.mx

**Keywords:** malaria, vector, drug resistance, scientific community

## Abstract

It is essential to establish a pattern to detect the strengths and weaknesses of working groups publishing on malaria, to promote coordination to facilitate the eradication of the disease. Given the complexity of the scientific network of groups and institutions studying malaria, it is necessary to use a mathematical algorithm that allows us to know the real structure of research on the disease in the world. In this work, articles with the word “malaria” in the title or author keywords gathered from Elsevier Scopus database were analyzed. By means of specific software, graphs were created. The analysis of the data allowed established different scientific communities, among which two were very diverse: one formed by those groups concerned about the vector transmission and control, and another one focused on the drug resistance of the parasite. Basic, applied, and operational research to eradicate malaria is an ambitious goal of the international institutions and the scientific community. The combination of effort and the establishment of a worldwide-scientific network that allows an effective interconnection (exchange) of knowledge, infrastructure technology, collaborators, financial resources, and datasets will contribute more effectively to end the disease.

## 1. Introduction

Malaria is one of the infectious diseases that generates the greatest concern to global health [1,2]. According to the last report of the World Health Organization, in 2016, malaria produced almost half a million deaths and more than 200 million cases were estimated, with 90% of these cases occurring in African countries. Malaria is a mosquito-borne disease caused by a parasite of the genus *Plasmodium*, with six species that affect humans: *Plasmodium falciparum*, which causes the most deaths, *Plasmodium vivax*, the most widely spread except in sub-Saharan Africa, *Plasmodium malariae*, *Plasmodium knowlesi*, *Plasmosdium ovale curtisi,* and *Plasmodium ovale wallikeri* [3,4,5,6].

*Plasmodium* has a complex life cycle, developing partially in mosquitoes of the genus *Anopheles* [7]. In humans, the parasite has a multiplicative exoerythrocytic stage, in hepatic cells, and another intraerythrocytic phase. The beginning of the cycle occurs with the bite of an infected female *Anopheles* mosquito, which inoculates the sporozoites in the vertebrate host. These migrate to hepatic cells, where they multiply and transform into exoerythrocytic merozoites that invade red blood cells again, generating erythrocytic merozoites that can invade new red blood cells and generate a new batch of merozoites or developed into gametocytes. Gametocytes will be ingested when *Anopheles* females bite the host to feed on their blood. In *P. vivax* and *P. ovale*, the hepatic schizont can remain dormant harboring hypnozoites that can be reactivated years later.

The malaria parasite is also genetically complex [8]. However, the current molecular tools make the analysis of genetic material available to produce data of great interest. The search for genes associated with drug resistance or phenotypes related to clinical manifestations of high pathogenicity, are two fundamental aspects addressed at a molecular level in malaria research. The nuclear genome of *P. falciparum* was sequenced in 2002 [6], and, later, the complete genomes of the rest of the human *Plasmodium* were obtained. These genomes have a size ranged from 23.3 to 33.6 Mb, organized into 14 chromosomes with a number of genes ranging from 5507 in *P. falciparum* to 7132 in *P. ovale curtisi*. In addition, *Plasmodium* parasites contain two more genomes. A mitochondrial genome, with the genes Cox1, Cox3 and Cytb [9], and an apicoplast genome, with genes related to the synthesis of fatty acids [10]. 

One of the most ambitious goals in current research on malaria is the search for a vaccine against the disease [11]. But, as already mentioned, the complex biology, genome, and life cycle of the parasite, as well as its ability to evade the protective immune responses of the host, have made the search unsuccessful. The design of malaria vaccines is very broad and varied. Currently, researchers are working with vaccines that act at different points in the *Plasmodium* life cycle. On the other hand, vaccines are being developed against a specific antigen, as well as vaccines against several antigens at different stages of the parasite lifecycle (multi-state vaccines), or even through the use of complete sporozoites. The use of viral vectors in heterologous prime-boost regimens represent another interesting alternative for the development of an effective vaccine. They are all the next-generation of malaria vaccines [12,13]. Other strategies being addressed in the fight against malaria focus on the use of mosquito nets and repellents to try to avoid mosquito bites [14].

The analysis of malaria research is an excessively complex system to carry out with a simple methodology. But, like other complex systems, it has a structure in scientific communities (SC), whose approach facilitates analysis [15]. The community detection is an issue that has been under study by the scientific community for a long time [16]. It is based on the search for substructures or communities that best represent the network’s topological features [17]. To obtain these substructures, it is common to use optimization techniques that combine the nodes in such a way that a certain objective function is maximized or minimized. In this regard, the modularity function has been successfully used by the scientific community to obtain a good definition of topologically related substructures. Modularity was proposed by Newman [18] and measures the quality of the cluster of nodes. The higher the value, the more precise the definition of the detected community structure. Since the invention of modularity, constant improvements have been generated [19] giving rise to one of the best-known algorithms: Girvan–Newman (GN), which is an algorithm based on a divisive method. This methodology has already been used successfully in many studies [20,21] and it is a very useful tool in bibliometric studies [22,23]. Thus, communities or clusters are generally groups of nodes that are more likely to be connected to each other than to members of other groups or to the rest of the network [24], in particular in bibliometric studies the scientific relations between groups of countries have been detected, or the topics around which a certain field of research is clustered [25,26]. Communities, in complex systems, can be defined as groups of nodes of a network, in which each node is densely connected with many other nodes in the network. The detection of communities is based on the principle that the nodes belonging to the same community are more likely to interact with each other. That is, they have greater connectivity. In this article, the authors introduce the novelty of using community detection to find SC associated with malaria research. This new approach will make it possible to establish future action policies in order to definitively eradicate malaria.

## 2. Materials and Methods 

The two large scientific databases, Web of Science (WoS) and Scopus, raise the major issue of comparison and consistency of the statistics derived from different data sources [27]. Various research studies have assessed the superposition between both, concluding that they are similar when the coverage since 1996 is restricted (Scopus coverage) [28,29]. Regarding the journals reported in the two large scientific databases such as Web of Science (WoS) and Scopus, a comparative analysis shows that the range of journals in WoS (13,605 journals) is smaller than that of Scopus (20,346 journals) [30], and the relationships between the results obtained with both databases for the number of articles and the number of citations obtained by countries, as well as for their rankings, are extremely strong (R^2^ ≈ 0.99) [31]. Therefore, only Scopus data have been used. This methodology has been used with success in different scientific fields [32,33,34,35].

**Figure 1:**
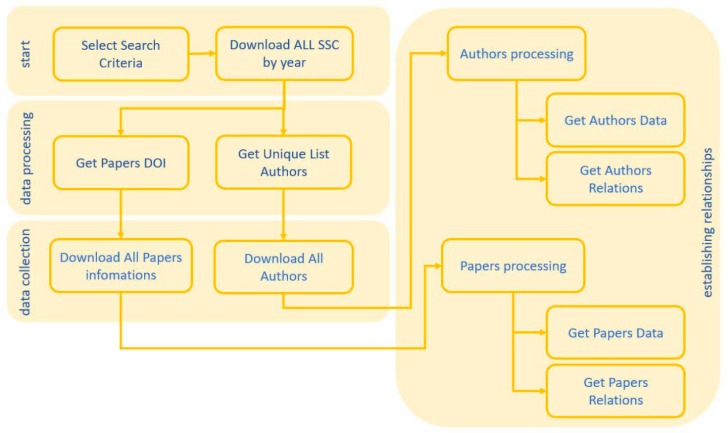
Methodology flowchart.

A software has been programmed for the massive download of data from Scopus using the developer application that allows this database. The SW can be divided into four functional blocks as described below, see Figure 1:

1. Start Module. Which requires as input the desired search criteria, for example: TITLE (“malaria”) OR AUTHKEY (“malaria”) OR ABS (malaria), once the criteria is selected, the time range of the search is required and a list of SCOPUS apikeys needed to use the Scopus search service (https://dev.elsevier.com/). The output (data files in json format containing the same data as when searching the web, with the difference that one file is obtained for every 25 search results) is the input for the next module of the application. It is worth highlighting the large volume of data files generated for a search criterion such as the one mentioned above.
2. Data processing. In this second stage, each one of the results obtained in the previous phase are analyzed, obtaining as output: a single list of the Scopus-ID of authors and a list of the DOIs of the articles.
3. Data collection. In this stage, two threads of work are launched in parallel: one that oversees downloading all the information of the authors for each one of the Scopus-ID of the list, generating at least one file per author; and another that will download all the information of the articles for each one of the DOIs of the list of papers. Therefore, a directory for authors and another for articles where all the mentioned information will be stored is obtained as output.
4. Establishing relationships. This module, like the previous one, is computationally more expensive and is subdivided in two: one for the analysis of the authors and the other for the analysis of the articles.
  - Authors collection. This submodule extracts all relevant information about the authors: H-Index, Name, Affiliation, Nation, etc. On the other hand, collaborations between authors are sought, for this purpose each author is examined, which are their articles, extracting all the authors from the papers of an author, establishing a bidirectional relationship between the author and the co-authors.
  - Papers collection. In this thread we obtain information about the articles, Keys, authors, co-authors, references to other papers, etc. For each paper we obtain a unidirectional relationship with the paper that cites, thus generating the network of the graph.

The Elsevier Scopus database was used to obtain information about published works on malaria. A complete search was performed using the search query: (TITLE-ABS-AUTHKEY (malaria)). The search range used was from 1900 to 2017. It should be noted that a different search query can give different results. To facilitate the analysis of the data, a powerful network analysis tool, Gephi, was used. The data extraction was carried out automatically through the implementation of a specific software called Research Network Bot (ResNetBot) [36]. This software allows the elaboration of a graph in which each publication on malaria is represented by a node, and the connections between two nodes represent the existence of a citation of one article on the other. With these data, a new graphic is constructed. This time, the nodes represent the researchers, while the relationships between the nodes represent the collaborations in at least one publication. Data obtained through ResNetBot was refined with the OpenRefine software (OR) (formerly Google Refine) (Google, Mountain View, CA, USA) and organized into spreadsheets to facilitate its management. The need to use OR is justified by the fact that authors often write slightly different keywords that identify the same concepts. In order to be able to unify these keywords OR is used because it provides the necessary mechanisms to find and merge written variations of the same word. E.g., “New York”, “New-York”, “new york”, etc. So, in our research the keywords need to be refined.

## 3. Results

### 3.1. Evolution of Scientific Output 

**Figure 2:**
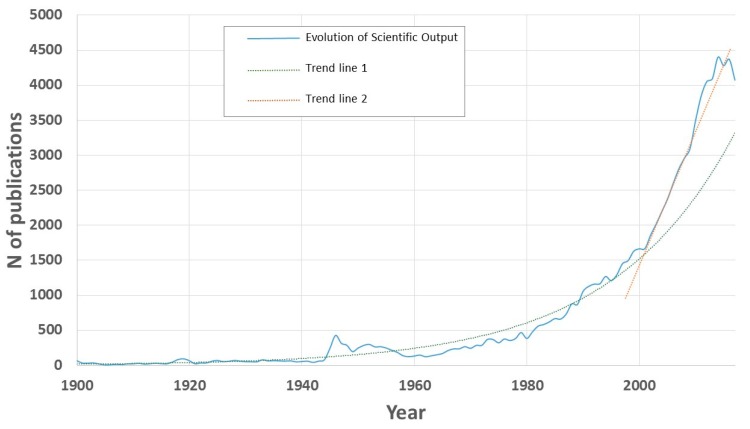
Publications trends on Malaria from 1900–2017.

The search yielded 85,370 results, whose growth is represented in Figure 2. As can be observed, the data show two trends. The first of them extends throughout the twentieth century, and it is exponential, while the second one is from the year 2000 to the present, and it is linear. In the first of the periods, the adjustment to the trend line is optimal, giving rise to a regression coefficient R^2^ greater than 0.9. The only data that separates from this trend line corresponds to the number of articles published in the year 1946, which is 429. This data is abnormally high. In fact, it is not reached and exceeded completely until the 1980s. The explanation for this is found in two milestones of great importance in the fight against malaria that took place in that year. On the one hand, chloroquine was recognized and established as an effective and safe antimalarial agent [37]. And, on the other hand, the CDC (Communicable Disease Center) was created from the Office of MCMA (Malaria Control in War Areas), whose main objective was to fight against diseases such as malaria in the South of the United States during the Second World War. Currently, the CDC is the main North American public health agency and its mission is “collaborate to create the expertise, information, and tools that people, and communities need to protect their health—through health promotion, prevention of disease, injury, and disability—and preparedness for new health threats.” Although the initial objective of the CDC was the control and elimination of malaria, its current role is aimed at prevention and surveillance, because it is considered eradicated in the USA since 1951.

The second period that can be observed in Figure 2 is adjusted to a linear trend line. Its slope is *m* = 159. That is, since 2000, each year, the average number of articles that have been published grows by more than 150 units. This highlights the enormous interest that research around malaria has, at present.

### 3.2. Authors and Countries in Malaria Research

**Figure 3:**
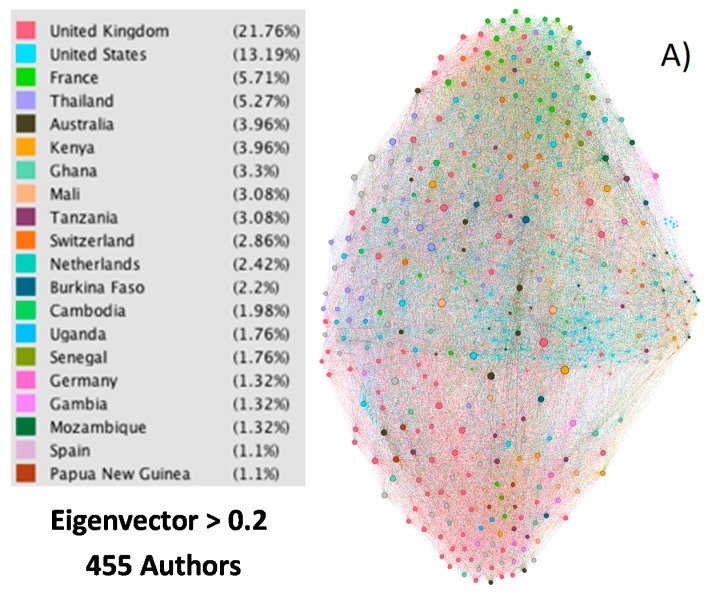

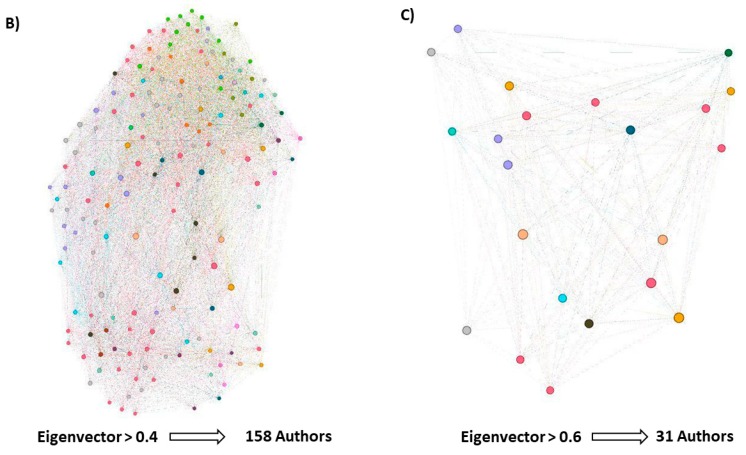
Graph representing by nodes the nationality of the authors with an eigencentrality value and their connections with the rest of the nodes (authors). (**A**) Greater than 0.2; (**B**) Greater than 0.4; (**C**) Greater than 0.6.

In the 85,370 published articles on malaria in the period studied, 148,876 authors and a total of 2,211,628 collaborations appear among them. In Table 1, the 20 most important authors can be observed. To establish this ranking, the eigenvector centrality or eigencentrality has been taken into account. In graph theory this is used to highlight the influence of a node in a network. Other values that could be used to measure the importance of an author are the H-index [38], the number of published articles, or the number of citations received by these articles. But the possibility that an author has worked in different areas or themes could mask the result. However, a high score of the eigenvector is directly related to the value that an author has within a network in which each author is identified with a node, and in which the connections of each node are preferably scored with other nodes of a high score. Figure 3A shows the 455 authors represented by nodes with an eigencentrality value greater than 0.2, and their connections with the rest of the nodes (authors). Figure 3B shows the 158 authors represented by nodes with an eigencentrality value greater than 0.4, and Figure 3C shows the 31 authors represented by nodes with an eigencentrality value greater than 0.6. The very high density of relations that exist between authors, it is possible to emphasize above all with an eigenvector >0.2. The size of each node is proportional to the value of its eigencentrality, and the color is representative of the country from which the author’s institutional affiliation. 

Figure 3 attempts to represent the relationship between authors from different countries, as well as their relevance. Therefore, a visualization (by color) of the authors from the different countries is sought, obtaining a general idea of which countries prevail over others, as well as the relevance of the main authors from one country versus authors from other countries. Due to the large number of nodes (authors) it is not possible to indicate their names. 

**Figure 4:**
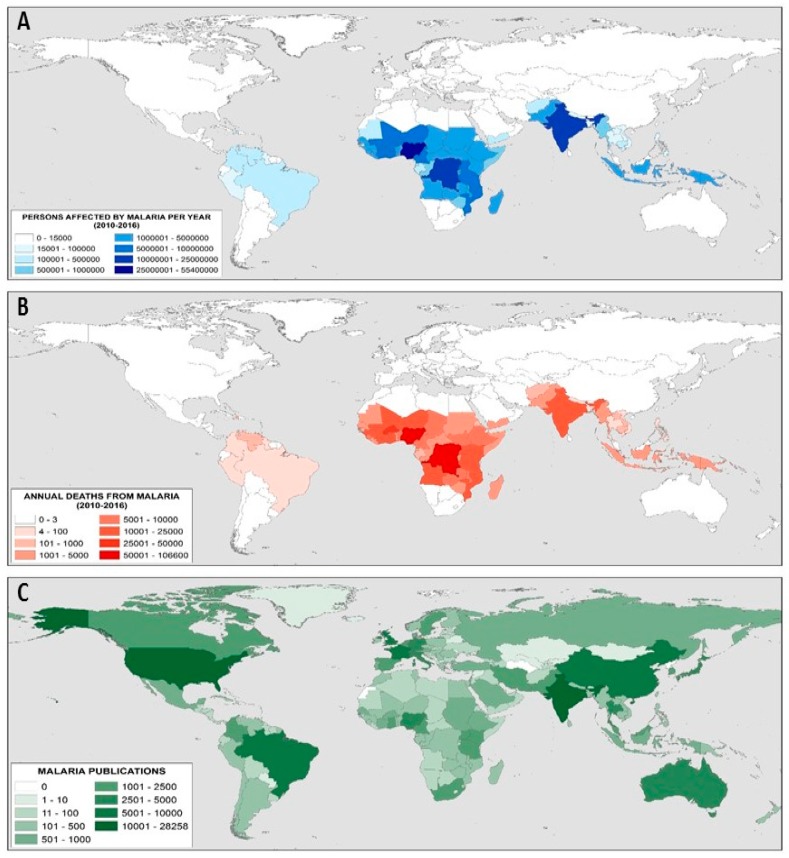
World map on malaria: (**A**) Cases estimated by the World Health Organization in the period 2010–2016; (**B**) Number of deaths estimated by the World Health Organization in the period 2010–2016; (**C**) Number of publications on malaria in the period 1900–2017.

As can be observed in Table 1, the first 20 authors in malaria research belong to ten different countries. These can be considered in two groups. On the one hand, the African (Kenya, Mali and Burkina Faso) and Asians (Thailand, Viet Nam and Laos) countries, of great importance for the number of estimated cases and deaths produced by malaria; and, on the other hand, the European (United Kingdom, and Netherlands), American (USA) and Australian (Australia) countries, of great importance for the number of articles published. Figure 4 shows the countries with the highest number of cases estimated by the World Health Organization in the period 2010–2016 (Figure 4A), which have presented a greater number of deaths estimated in that same period (Figure 4B), and those that have published the most articles in 1900–2017 (Figure 4C). Burkina Faso and Mali are among the five countries with the highest number of deaths, while the United States and the United Kingdom are the two countries whose institutions publish the most articles on malaria. For this reason, it is not surprising that the main researchers working in these countries are those with the highest eigencentrality.

### 3.3. Communities Detection 

**Figure 5:**
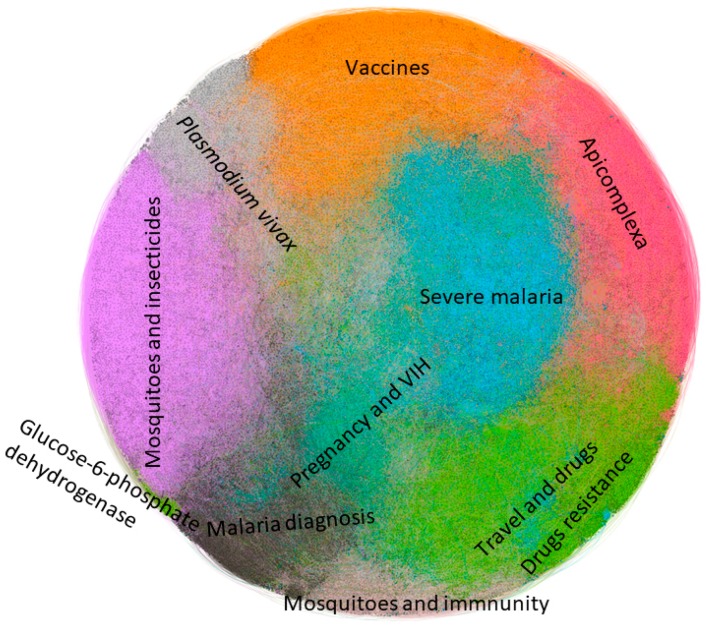
Graph representing the 11 main scientific communities.

From 85,370 published articles, 714,979 citations were counted—the citations to nodes of topics other than malaria were not included. On the other hand, 149,143 keywords defined by the authors appear in these articles. Both data allow us, using genetic algorithms, to establish a series of thematic SC, of which, the most frequent are represented in Table 2. In the table only appear SC composed of at least 1% of the total of 85,370 articles published. To supplement Table 2, Figure 5 has been carried out. The information that codifies Figure 5 has to do with the relative size of one community in front of the other as well as the proximity of certain communities to each other, depending on how close the nodes in the graph are.

Keywords 1 to 5 appear ordered according to their appearance frequency in each SC, after eliminating malaria as keyword, since it appears in first place in all detected communities, but its presence does not contribute anything to the analysis. In Figure 5, the 11 main SC appear highlighted using different colors.

Communities #1 and #2 had the highest proportion of published articles, and each of them is focused on the two main fields of action that currently exist against malaria: the fight against mosquitoes and drug resistance. Today, it is demonstrated that the most effective way to reduce severe malaria and the number of deaths caused by the disease, in endemic regions, is the use of insecticide-treated bed nets (ITNs) [39,40]. The use of ITNs reduces both the number of mosquitoes and their length of life, producing a double protection effect in the affected regions. The only class of insecticides allowed in the ITNs are the pyrethroids, due to the low toxicity effect in human health, and to their slow decomposition. Therefore, it is essential to find new systems that prevent the transmission of malaria, as well as new insecticides that repel mosquitoes from areas of human concentration. The second major concern of the international scientific community in the fight against malaria is the presence of drug resistance in *Plasmodium*. Over time, as new antimalarial drugs have been introduced, the parasite has become resistant to them. First, it happened with chloroquine and other similar 4-aminoquinolines. Then, resistance to sulfadoxine-pyrimethamine. And, more recently, cases of parasites resistant to drugs derived from artemisinin have also been discovered [41]. So, the long-term success of this strategy will depend to a large extent on the control over the ways that the parasite develops drug resistance. Therefore, it is a priority to approach this problem from different perspectives. It will have to be done both from the pharmacological point of view, with the use of drug combinations with different formulations, and from the strategic point of view, in terms of health infrastructure.

The following SC #3, #4, #5, and #6 are focused on the study of apicomplexa, severe malaria, diagnosis and vaccines, respectively. Of them, the research on a vaccine that ends the disease arouses the greatest interest [42]. There is currently no effective malaria vaccine, but there are three lines of action targeting key points in the life cycle of the malaria parasite: the anti-infection approach (pre-erythrocytic vaccines), blood-stage vaccines, and transmission-blocking vaccines, interrupting the spread of infection. It is likely that the definitive solution comes from the combination of several of these approaches. At the moment, one pre-erythrocytic candidate, the RTS,S vaccine [43], is going to be administered in a pilot implementation. 

The last five communities of Table 2—SC #7, #8, #9, #10, and #11—involve topics such as pregnancy and VIH, *Plasmodium vivax*, mosquitoes and immunity, travel and drugs, and glucose-6-phosphate dehydrogenase. Of these, research in *P. vivax*, a highly prevalent parasite in most affected areas except Africa, is becoming very important. Although more severe cases of malaria associated exclusively with *P. vivax* are detected, a large number of mixed infections of *P. falciparum* and *P. vivax* are diagnosed in patients living in areas where both species are prevalent. For the rest, it should be borne in mind that pregnant women and their unborn children are particularly vulnerable to the disease, that every year there are detected thousands of cases of malaria in travelers in countries where the disease was eliminated, and there is a concern about the use of primaquine in people with a deficiency in glucose-6-phosphate dehydrogenase (G6PD), as it can cause severe hemolysis. All these aspects focus the research of a large number of work teams throughout the world.

## 4. Discussion

### 4.1. Findings

Eradication of malaria is on the global health agenda. Therefore, now is the time to ask ourselves at what point malaria stands, how much we know about the human disease, the parasite and the mosquito vectors [44,45] and take the correct directions for malaria control and elimination.

After more than a century of research on malaria, the number of articles published on it is enormous. There are almost 100,000 records, with different approaches and an accumulated knowledge acquisition that makes necessary an analysis of the data based on algorithms that facilitate its interpretation.

After analyzing about 150,000 authors and the more than two million relationships established between them, we have been able to establish a parameter that measures the role played by each author in the complex network of researchers who published on malaria research. So, we have seen that authors like Kevin Marsh (Kenya Medical Research Institute, Kenya), Abdoulaye A. Djimde (University of Sciences, Department of Epidemiology of Parasitic Diseases, Mali), Chris J. Drakeley (London School of Hygiene & Tropical Medicine, United Kingdom), or Ogobara K. Doumbo (University of Bamako Faculty of Medicine, Mali), occupy a position of enormous relevance. The topics of their research work range from the characterization of malaria as a severe disease, for Marsh et al., to the control of transmission, for Drakeley et al., or the study of drug resistance, for Djimde et al. But all of them have in common a very high consideration on the part of their colleagues and an extensive network of collaboration that favor the advance of the malaria knowledge. 

A second aspect that we have considered is the set of keywords with which the authors delimit and define the scope of their research. The analysis of the 150,000 keywords appeared in the articles on malaria has allowed us to detect, through the use of an iterative algorithm, up to 11 communities. The analysis of these communities allows us to draw a map about worldwide malaria research. Thus, we have seen that most research efforts are focused on drug resistance and control of the mosquito vector species. Also, of great importance, especially in recent years, the research focused on vaccine development or knowledge of parasites other than *Plasmodium falciparum*, such as *P. vivax*.

### 4.2. Limitations

In such a large data analysis, as that carried out for all published works on malaria, an additional problem is that of the dense data representation, it has been found that representation values as a function of eigenvector value >0.2 is appropriate. 

### 4.3. Future Work

After eradicating malaria from Europe, North America, the Caribbean, and some countries of South-Central America and Asia, the time has come to consider removing it from the countries where, at present, malaria is a true public health problem, such as the sub-Saharan Africa countries. But, for this, the combination of all the tools with which science counts nowadays will be necessary. The collaborative networks between countries and scientists of notable importance will have to be strengthened, so that, the confluence of their knowledge finds the end to malaria. No one is thinking that a simple approach will eliminate malaria in Africa or other areas. It will not be just the fight against the mosquito, or the administration of a vaccine. It will be the union of all efforts in the search for a global strategy, also at a political, financial, educational, and social level.

## 5. Conclusions

These results allow us to have a global view of the state of world malaria research. This is the first time that a manuscript studies the relationship between the work of the most important groups in this field. For the first time, the lines of research of greatest interest to the international scientific community have been drawn, and the multilateral relations that are taking place between the different scientific communities involved in this research have been established. Furthermore, this study shows that the role of each scientist, each institution or each country in the fight to eradicate malaria is unique and plays a unique role within the network of which it forms a part.

## Figures and Tables

**Table 1:** List of authors most frequent in malaria research articles.

Author	Eigencentrality	N_m_	N_t_	H-Index	Coauthor	Cites	Country
Marsh K.	1.000000	392	468	84	1748	26032	Kenya
Djimde A.	0.995692	120	123	33	1052	4656	Mali
Drakeley C.	0.965505	284	294	58	1781	11934	United Kingdom
Doumbo O.	0.957462	389	424	56	1921	13477	Mali
Nosten F.	0.829049	512	568	78	2160	23142	Thailand
Ouédraogo J.	0.816295	119	147	29	962	3137	Burkina Faso
Borrmann S.	0.816208	87	87	31	676	3818	Kenya
Mueller I.	0.808423	276	289	40	1224	5830	Australia
Price R.	0.786288	227	241	54	1150	9966	United Kingdom
Hien T.	0.786204	246	271	70	1763	19209	Viet Nam
Plowe C.	0.749014	179	187	55	1216	10896	United States
D’Alessandro U.	0.746263	298	315	43	1483	7497	United Kingdom
White N.	0.726106	897	1146	114	3134	52466	Thailand
Mayxay M.	0.703699	113	117	32	866	3941	Laos
Bousema T.	0.702141	136	165	34	827	4205	United Kingdom
Kwiatkowski D.	0.690173	270	335	63	2069	17684	United Kingdom
Dondorp A.	0.685832	285	312	52	1367	10554	Netherlands
Ogutu B.	0.681686	130	139	27	1017	3031	Kenya
Fanello C.	0.680121	42	43	21	508	2648	Thailand
Greenwood B.	0.676612	458	893	77	1848	23579	United Kingdom

Nm, number of articles published on malaria; Nt, number of total articles published, including those whose main topic is not malaria; Coauthor, number of co-authors that appear in articles published on malaria; Cites, number of citations that have received articles published on malaria.

**Table 2:** Main scientific communities in malaria research.

Comm.	%	Community Topic	Keyword 1	Keyword 2	Keyword 3	Keyword 4	Keyword 5
#1	5.64	Mosquitoes and insecticides	*An. gambiae*	*Anopheles*	*P. falciparum*	Malaria vector	Vector control
#2	5.40	Drug resistance	*P. falciparum*	Artemisinin	Chloroquine	Drug resistance	Antimalarial
#3	3.28	Apicomplexa	*P. falciparum*	*Plasmodium*	*P. berghei*	Apicoplast	Erythrocyte
#4	3.16	Severe malaria	*P. falciparum*	Cerebral malaria	Cytokines	Severe malaria	Children
#5	3.13	Malaria diagnosis	*P. falciparum*	Diagnosis	Microscopy	*Plasmodium*	*P. vivax*
#6	3.05	Vaccines	*P. falciparum*	Vaccine	*Plasmodium*	*P. vivax*	Malaria vaccine
#7	1.77	Pregnancy and VIH	Pregnancy	*P. falciparum*	HIV	Placenta	Anemia
#8	1.44	*Plasmodium vivax*	*P. vivax*	*P. falciparum*	Primaquine	Chloroquine	Thrombocytopenia
#9	1.35	Mosquitoes and immunity	Mosquito	*P. falciparum*	*Plasmodium*	*An. gambiae*	*Anopheles*
#10	1.31	Travel and drugs	*P. falciparum*	Mefloquine	Chemoprophylaxis	Travel	Prophylaxis
#11	1.03	Glucose-6-phosphate dehydrogenase	*P. falciparum*	G6PD deficiency	Oxidative stress	Thalassemia	Sickle cell disease

Comm., community; An. *Anopheles*; *P. Plasmodium*. %, percentage of articles belonging to a community with respect to the total number of published articles in malaria.
